# Artificial Intelligence Provides Accurate Quantification of Thoracic Aortic Enlargement and Dissection in Chest CT

**DOI:** 10.3390/diagnostics14090866

**Published:** 2024-04-23

**Authors:** Nicola Fink, Basel Yacoub, U. Joseph Schoepf, Emese Zsarnoczay, Daniel Pinos, Milan Vecsey-Nagy, Saikiran Rapaka, Puneet Sharma, Jim O’Doherty, Jens Ricke, Akos Varga-Szemes, Tilman Emrich

**Affiliations:** 1 Division of Cardiovascular Imaging, Department of Radiology and Radiological Science, Medical University of South Carolina, Charleston, SC 29425, USA; 2 Department of Radiology, University Hospital, LMU Munich, 81377 Munich, Germany; 3 Medical Imaging Center, Semmelweis University, Korányi Sándor utca 2, 1083 Budapest, Hungary; 4 Heart and Vascular Center, Semmelweis University, Varosmajor utca 68, 1122 Budapest, Hungary; 5 Siemens Healthineers, Princeton, NJ 08540, USA; saikiran.rapaka@siemens-healthineers.com (S.R.); sharma.puneet@siemens-healthineers.com (P.S.); 6 Siemens Medical Solutions, Malvern, PA 19355, USA; james.odoherty@siemens-healthineers.com; 7 Department of Diagnostic and Interventional Radiology, University Medical Center of the Johannes Gutenberg-University, Langenbeckstr. 1, 55131 Mainz, Germany; 8 German Centre for Cardiovascular Research, 55131 Mainz, Germany

**Keywords:** artificial intelligence, deep learning, computed tomography, aortic aneurysm, aortic dissection

## Abstract

This study evaluated a deep neural network (DNN) algorithm for automated aortic diameter quantification and aortic dissection detection in chest computed tomography (CT). A total of 100 patients (median age: 67.0 [interquartile range 55.3/73.0] years; 60.0% male) with aortic aneurysm who underwent non-enhanced and contrast-enhanced electrocardiogram-gated chest CT were evaluated. All the DNN measurements were compared to manual assessment, overall and between the following subgroups: (1) ascending (AA) vs. descending aorta (DA); (2) non-obese vs. obese; (3) without vs. with aortic repair; (4) without vs. with aortic dissection. Furthermore, the presence of aortic dissection was determined (yes/no decision). The automated and manual diameters differed significantly (*p* < 0.05) but showed excellent correlation and agreement (r = 0.89; ICC = 0.94). The automated and manual values were similar in the AA group but significantly different in the DA group (*p* < 0.05), similar in obese but significantly different in non-obese patients (*p* < 0.05) and similar in patients without aortic repair or dissection but significantly different in cases with such pathological conditions (*p* < 0.05). However, in all the subgroups, the automated diameters showed strong correlation and agreement with the manual values (r > 0.84; ICC > 0.9). The accuracy, sensitivity and specificity of DNN-based aortic dissection detection were 92.1%, 88.1% and 95.7%, respectively. This DNN-based algorithm enabled accurate quantification of the largest aortic diameter and detection of aortic dissection in a heterogenous patient population with various aortic pathologies. This has the potential to enhance radiologists’ efficiency in clinical practice.

## 1. Introduction

Thoracic aortic aneurysms (TAAs) frequently remain asymptomatic [1] and are typically diagnosed either as an incidental finding or due to severe complications, such as aortic dissection or rupture [2]. Given the high mortality associated with such acute aortic events [3,4], early detection and adequate follow-up of aortic dilatation and aneurysm are crucial to prevent life-threatening complications.

Accurate measurements of the aortic diameter are required to confirm a diagnosis and to adequately treat and monitor patients with known TAA or aortic dilatation and should be performed perpendicular to the long aortic axis [5]. However, aortic enlargement is a common missed finding in computed tomography (CT) scans [6], particularly if the examination was performed due to another clinical question and unrelated indication. Even in cases of known aortic dilatation or aneurysm, accurate follow-up measurements remain tedious and time-consuming and suffer from high inter- and intrareader variability [7]. In this context, artificial intelligence (AI) algorithms for automated segmentation and measurements of the aorta have already been shown to reduce the number of missed findings in CT scans [8] and to increase their inter- and intrareader agreement while significantly reducing the reporting times [9]. Recent advancements in deep learning have shown promising results in segmenting and quantifying aortic diameters, even in cases with altered vascular architecture, e.g., due to aneurysms or dissections [10]. Most studies have evaluated automated aortic diameter measurements at predefined landmarks according to the recommendations given by the American Heart Association (AHA) [2] and excluded patients with aortic pathologies such as aortic dissection or after aortic repair. However, as the maximum aortic diameter may not align precisely with those predefined measurements, the complete extent of the disease may remain hidden if measurements are performed at the predefined landmarks only. Furthermore, the performance of such AI algorithms might be influenced by other pathological aortic findings, such as postoperative changes after aortic repair, which are commonly observed in patients undergoing CT-based assessment of the thoracic aorta.

Therefore, this study aimed to assess the performance of a deep neural network (DNN)-based AI algorithm in quantifying the thoracic aortic diameter at the most dilated location in non-contrast and contrast-enhanced chest CT, as well as detecting aortic dissections, each in a heterogenous population with various aortic pathologies.

## 2. Materials and Methods

### 2.1. Study Population

The local institutional review board approved this retrospective, single-center, Health Insurance Portability and Accountability Act (HIPAA)-compliant study and waived requirement for informed consent. Patients were identified from the institutional vascular surgery registry using the international classification of diseases (ICD-10) codes for thoracic and thoracoabdominal aortic aneurysm. All the included patients underwent non-contrast and contrast-enhanced (arterial contrast phase) electrocardiogram (ECG)-gated CT scans of the thoracic aorta. Patients younger than 18 years old and/or with missing non-enhanced scans were excluded from this study. Demographic and clinical patient characteristics including history of prior aortic repair were collected from their medical records. In order to evaluate the algorithm’s performance in different scenarios and how this may potentially impact its accuracy, subgroup analysis was conducted as follows: (I) ascending aorta vs. descending aorta, (II) non-obese vs. obese patients (body mass index [BMI] < vs. ≥30 kg/m^2^), (III) patients without vs. with prior aortic repair and (IV) patients without vs. with aortic dissection.

### 2.2. Image Acquisition and Reconstruction

The CT scans were acquired using three different systems (SOMATOM Force, Definition AS+, and Emotion 16, Siemens Healthineers, Forchheim, Germany) and performed according to the institutional standard protocols, with prospective ECG triggering at 70% of the R-R interval at heart rates under 70 beats per minutes (bpm) and at 40% for heart rates over 70 bpm. All the patients received a non-enhanced scan, followed by a contrast-enhanced scan using an iohexol iodinated contrast (Omnipaque 350, GE Healthcare, Waukesha, WI, USA).

### 2.3. Convolutional Neural Network Model and Measurements

**Figure 1:**
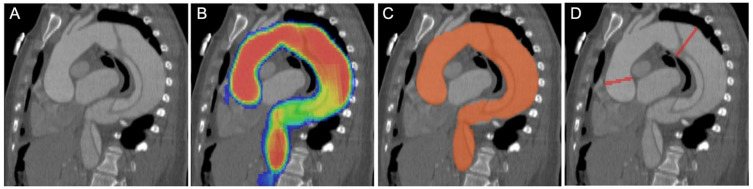
Steps involved in quantifying diameters at the widest location in each section of the aorta. (**A**) Candy-cane view of a thoracic aorta with a dissection in contrast-enhanced CT. (**B**) Probability map of the AI prediction model of the aorta. The red color shows the highest confidence for the model. Yellow, green and blue each represent a decreasing level of confidence, respectively, with red indicating the highest confidence. (**C**) An inclusion cutoff for the confidence level is picked, and the segmentation of the aorta can be generated based on this selection. (**D**) From this segmentation, the maximum diameters in each section may be calculated. AI = artificial intelligence; CT = computed tomography.

**Figure 2:**
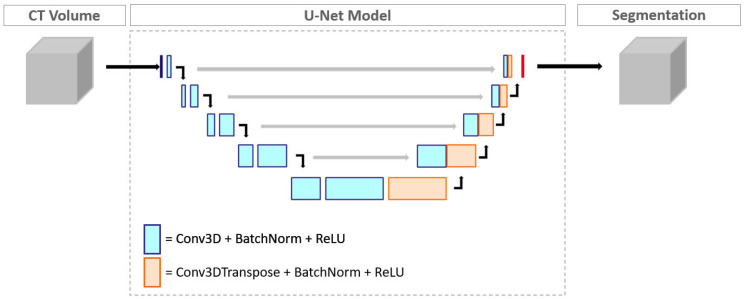
Architecture of the U-Net segmentation model.

The aortic diameter measurements were performed using a combination of multiple DNN models (Figure 1). An image-to-image U-Net segmentation model was first used to segment the aortic region (Figure 2). Then, a deep reinforcement-learning-based landmark model was used to detect landmarks corresponding to the aortic root, brachiocephalic artery, left common carotid artery, left subclavian artery and celiac artery. These landmarks and the aortic segmentation were used to compute the aortic centerline, along with a local coordinate system denoting the normal and in-plane directions (defining the cross-sectional plane) to the centerline at each point. The length of the aortic centerline was then partitioned into regions corresponding to the ascending, aortic arch and descending sections of the aorta, and the maximum average diameters in the ascending and descending diameter are given as outputs.

The aorta segmentation model was trained on a dataset comprising 1582 CT images of the chest, including contrast-enhanced and non-contrast enhanced images. The region corresponding to the aorta in each image was annotated manually by trained human readers on axial slices, and the smoothness and consistency of the annotation were verified in the coronal and sagittal views. Each annotation was manually verified by a radiologist for accuracy before being added to the training dataset, taking a total time of 2–4 h per dataset depending on the number of slices in the image and the presence of any aortic pathologies. The aorta segmentation model has a U-Net architecture, utilizing encoder blocks with 3 × 3 × 3 convolutional kernels, along with batch normalization and rectified linear (ReLU) activation functions. The network has a depth of 5 layers, with the number of filters doubling at each layer in combination with a corresponding reduction in the image size. The model was trained using the Adam optimizer with an initial learning rate of 10^−4^ and the standard cross entropy loss function. Data augmentation strategies such as randomized window settings and the addition of noise to the images were used to improve the generalizability of the model, and the input volume was resampled to a uniform resolution of 2 mm in each spatial direction before being fed into the network. A subset of 20% of the total annotated data was used as the validation dataset, on which the trained model achieved an average Dice coefficient of 0.93 ± 0.03.

The deep reinforcement-learning-based landmark model learns to identify anatomically relevant point landmarks in medical images [11]. To summarize, the model trains computational agents which look at the image at different spatial resolutions and learn to navigate the image from randomly chosen initial locations to the target landmark location. The agents are trained by assigning appropriate rewards to the agent in each run when they hit the landmark target. For our application, the different landmark agents were trained at image resolutions of 2, 4, 8 and 16 mm. On an independent validation dataset, the aorta landmark models achieved an average accuracy of 4.5 mm in the Euclidean distance from the target anatomical landmark.

Once the aorta segmentation and centerline were obtained, the dataset was reformatted into a simple rectangular volume stretched along the centerline of the aorta, with an in-plane resolution of 1.0 mm and a spacing of 2.0 mm between different cross-sectional slices. A simple 2D model extracted the features along each cross-sectional slice, which were then passed to a Support Vector Machine (SVM)-based classifier using a radial-basis kernel. The SVM classifier outputs the probability of each slice containing an aortic dissection, and the probabilities along all the slices are then pooled together to make a patient-level prediction of the presence of dissection.

### 2.4. Image Reading

The images were evaluated independently by a board-certified radiologist and a radiology resident with 13 and 2 years of experience in cardiovascular imaging, respectively, using a dedicated workstation (Aquarius Intuition Edition v4.4.12, TeraRecon, Inc., Foster City, CA, USA). The readers were blinded to the original radiology report in an independent reading with the patients in random order. Radiological reading was performed using the contrast-enhanced scans, and the non-enhanced scans were used only for the AI-based analysis. This radiological reading was independent of the AI results. The contrast-enhanced scans were assessed by measuring the aortic diameter in multiplanar reformat (MPR) images perpendicular to the vessel centerline at the largest dilation in both the ascending and descending thoracic aorta, using an automated edge detection tool. Manual adjustments of the automated edge detection were performed if necessary. The ascending thoracic aorta was defined as extending from the aortic annulus to the origin of the brachiocephalic artery, while the descending thoracic aorta was considered from the left subclavian artery to the diaphragm [5]. In addition, readers evaluated the scans regarding the presence of aortic dissection (yes/no decision).

### 2.5. Statistical Analysis

The statistical analysis was performed and graphically illustrated using GraphPad Prism (Version 8.4.2; GraphPad, San Diego, CA, USA) and RStudio (Version 1.4.1717, RStudio Inc., Boston, MA, USA). The Kolmogorov–Smirnov test was used to test the continuous variables for normality. Depending on the normality distribution, continuous data are reported as means ± standard deviation (SD) or as medians with interquartile ranges. Categorial variables were described as absolute frequencies and proportions. *p* values below 0.05 were considered statistically significant.

Two-way, mixed-effects, single-rater intraclass correlation (ICC) with absolute agreement was used to analyze the inter-reader agreement and was interpreted as follows: <0.5 poor agreement; 0.5–0.75 moderate agreement; 0.75–0.9 good agreement; >0.9 excellent agreement [12].

The manual and automated measurements were compared overall, as well as in the following subgroups: (I) ascending aorta vs. descending aorta, (II) non-obese vs. obese patients (body mass index [BMI] < vs. ≥30 kg/m^2^), (III) patients without vs. with prior aortic repair, (IV) patients without vs. with aortic dissection.

The Wilcoxon matched-pairs signed rank test was used to compare the automatically derived diameters to those from the manual measurements and to compare the errors between subgroups. Spearman’s correlation was used to assess the correlation between the automated and manual measurements. The agreement was analyzed using Bland–Altman analysis (mean bias, upper/lower limits of agreement [LoA]) and two-way, mixed-effects, single-rater ICC with absolute agreement, as described above.

The absolute (magnitude of the difference between the automated and manual measurements without considering its direction) and systematic errors (deviations, including over- and underestimation) between the automated and manual values were analyzed.

For the performance analysis of AI-based aortic dissection detection, its sensitivity, specificity and accuracy are reported, each with a 95% confidence interval (CI).

## 3. Results

### 3.1. Patient Population

A total of 100 patients (median age 67.0 [55.3/73.0] years; 60.0% male) with TAA or dilation of the ascending and/or the descending thoracic aorta were included in this study. The ascending aorta was predominantly affected in 44, while the descending aorta was affected in 56 patients. Nearly two-thirds of the patients (*n* = 62) had pathologic findings in their thoracic aorta due to prior repair (surgical, *n* = 29; endovascular, *n* = 17; both, *n* = 10) and/or aortic dissection (*n* = 42). The clinical patient characteristics are shown in Table 1. All the patients underwent non-enhanced and contrast-enhanced chest CT, resulting in a total of 200 scans being included. Three non-enhanced and two contrast-enhanced scans could not be processed by the DNN.

### 3.2. Aortic Diameter Quantification in the Entire Cohort

**Figure 3:**
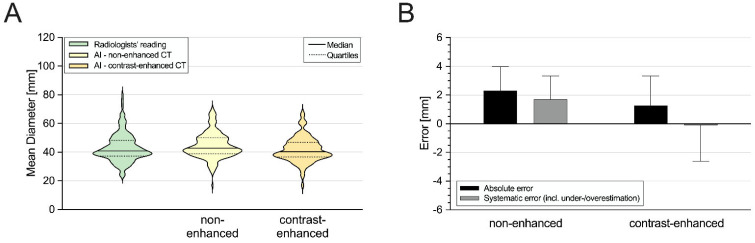
(**A**) Comparison between manual and automated overall aortic diameters as well as (**B**) absolute and systematic errors in automated vs. manual measurements. AI = artificial intelligence; CT = computed tomography; incl. = including.

Since the inter-reader agreement was excellent (ICC = 0.97), all the analyses were conducted using the average of the manual measurements taken by the two readers. Table 2 and Figure 3 illustrate the detailed results for the overall aortic diameter quantification comparing between the manual and automated measurements.

The manually acquired diameters significantly differed from the automated measurements derived for the non-contrast (40.9 mm [37.3–48.2] vs. 42.9 mm [38.9–50.0]; *p* < 0.05) and the contrast-enhanced scans (40.9 mm [37.3–48.2] vs. 40.3 mm [36.6–46.9]; *p* < 0.05). The non-contrast AI-based values were significantly higher than the corresponding contrast-enhanced values (*p* < 0.05). The absolute and relative errors compared to the manual measurements were lower in the AI-based diameters from the contrast-enhanced scans than those from the non-contrast scans (absolute error: 2.3 mm [1.3–4.0] vs. 1.3 mm [0.5–3.3]; systematic error: 1.7 mm [−0.3–3.3] vs. −0.1 mm [−2.6–0.8]; each *p* < 0.05; Figure 3B). There was a strong correlation and excellent agreement between the manual and automated measurements (non-contrast: r = 0.86, ICC = 0.93; contrast-enhanced: r = 0.89, ICC = 0.94).

**Figure 4:**
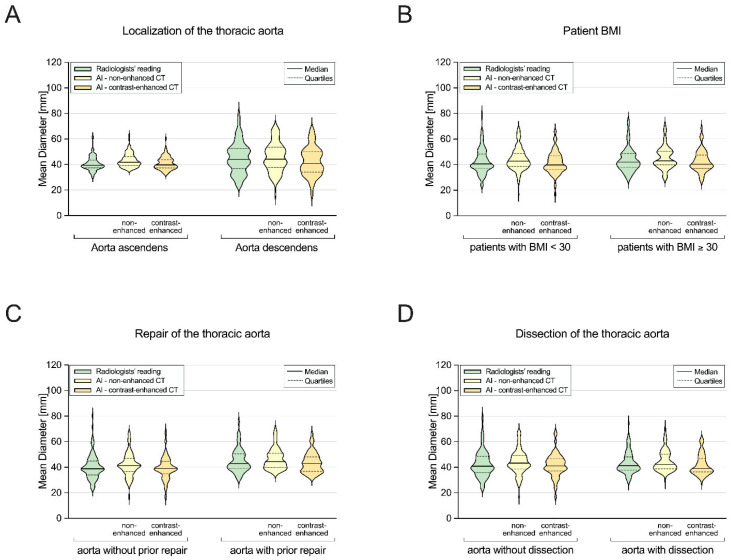
Comparison between manual and automated aortic diameters in the following subgroups: (**A**) ascending aorta vs. descending aorta; (**B**) non-obese vs. obese patients; (**C**) patients with vs. without prior aortic repair; (**D**) patients with vs. without aortic dissection. AI = artificial intelligence; BMI = body mass index; CT = computed tomography; incl. = including.

**Figure 5:**
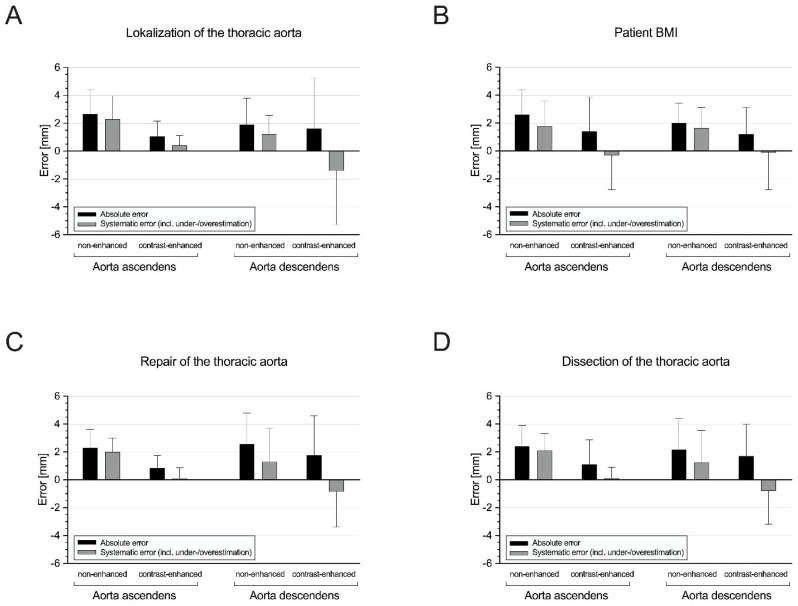
Absolute and systematic errors of automated vs. manual measurements in the following subgroups: (**A**) ascending aorta vs. descending aorta; (**B**) non-obese vs. obese patients; (**C**) patients with vs. without prior aortic repair; (**D**) patients with vs. without aortic dissection. AI = artificial intelligence; BMI = body mass index; CT = computed tomography; incl. = including.

Further subgroup analysis was performed using the contrast-enhanced images since the manual reading was also conducted using the contrast-enhanced scans. However, the AI-based results from the non-contrast scans are additionally illustrated in Figure 4 and Figure 5.

### 3.3. Subgroup I: Ascending Aorta vs. Descending Aorta

While the manual and automated measurements were similar in the ascending aortas (39.5 mm [37.4–43.3] vs. 39.8 mm [37.4–43.9]), they were significantly different in the descending aortas (43.8 mm [36.3–52.5] vs. 40.8 mm [34.1–50.0]; *p* < 0.05) (Figure 4A). The AI-based diameters of the ascending aortas showed lower absolute and systematic errors (each *p* < 0.05; Figure 5A), as well as a lower mean bias (0.3 [LoA, −5.3/5.9] vs. −3.0 [LoA, −12.7/6.7]). However, the automated diameters of the descending aortas showed a slightly higher correlation and agreement with the manual diameters (Table 2).

### 3.4. Subgroup II: Non-Obese vs. Obese Patients

The manual and automated diameters significantly differed in the non-obese patients (40.4 mm [37.3–48.2] vs. 39.8 mm [36.3–46.8]; *p* < 0.05) but were similar in the obese patients (42.1 mm [37.8–48.6] vs. 40.4 mm [37.1–47.5]) (Figure 4B), with a slightly higher mean bias (Table 1) and systematic error rate in the non-obese than in the obese patients (*p* < 0.05; Figure 5B). Nevertheless, the automated measurements showed strong correlation and excellent agreement with the manual diameters in both subgroups (Table 2).

### 3.5. Subgroup III: Patients without vs. with Prior Aortic Repair

The AI-based and manually measured values were similar in the patients without prior aortic repair (38.9 mm [33.9–44.8] vs. 39.1 mm [34.9–44.6]) but significantly different in the patients with aortic repair (43.0 mm [39.0–50.5] vs. 43.0 mm [36.8–48.2]; *p* < 0.05) (Figure 4C). Nevertheless, the patients without and with prior aortic repair showed a similar level of systematic error (*p* = 0.826; Figure 5C), as well as a high correlation and excellent agreement between the automated and manual measurements (Table 2).

### 3.6. Subgroup IV: Patients without vs. with Aortic Dissection

While the manual and automated obtained diameters were similar in the patients without aortic dissection (40.9 mm [35.7–48.5] vs. 41.1 mm [37.1–46.8]), they were significantly different in those with aortic dissection (41.4 mm [37.7–48.2] vs. 39.0 mm [36.5–46.9]; *p* < 0.05) (Figure 4D), with a significantly higher systematic error in rate the patients with aortic dissection (*p* < 0.05; Figure 5D). Nevertheless, the AI-based diameters showed a high correlation and excellent agreement with the manual values in both the patients with and without aortic dissection (Table 2).

**Figure 6:**
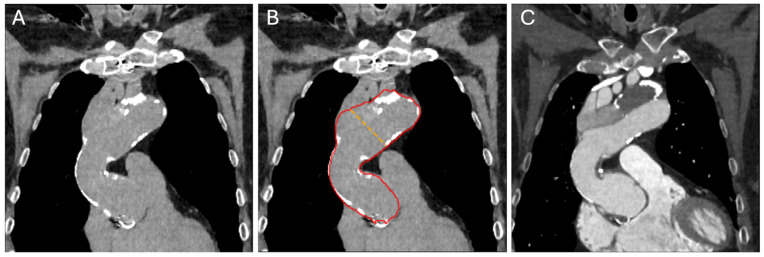
Seventy-two-year-old woman with a medical history of open surgical repair of ascending aortic aneurysm with graft placement and development of chronic dissection. (**A**) Non-enhanced scan as basis for (**B**) aortic borderline (red line) detection by the algorithm demonstrating accurate segmentation of the aorta including both the true and false lumen. (**C**) Contrast-enhanced scan with visible aortic dissection and dilation.

Figure 6 illustrates a sample case of accurate AI-based aortic segmentation even in a non-enhanced scan of a patient with a history of open surgical aortic repair and chronic dissection.

### 3.7. DNN-Based Detection of Aortic Dissection

The accuracy, sensitivity and specificity of DNN-based aortic dissection detection were 92.1% (95% CI: 84.5–96.8%), 88.1% (95% CI: 74.4–96.0%) and 95.7% (95% CI: 85.5–99.5%), respectively.

## 4. Discussion

This study assessed the performance of a DNN algorithm for automated measurements of the thoracic aorta diameter in non- and contrast-enhanced chest CT scans, as well as automated detection of aortic dissection. The main findings are as follows: First, the overall automated measurements showed a strong correlation and excellent agreement with the corresponding manually derived values. Second, similar results were observed in the different subgroups depending on the anatomic aortic segment, the patient’s BMI and the presence of prior aortic repair and/or dissection. Third, this algorithm accurately detected the presence of aortic dissection.

Dilation of the thoracic aorta is often asymptomatic but at the same time represents a potentially life-threatening condition. The aortic diameter and its growth rate over time are the main predictors of fatal complications, such as aortic dissection and rupture [5], therefore playing a crucial role in patient monitoring before and after aortic repair. However, manual aortic measurements in CT are challenging, time-consuming and prone to intra- and intra-reader variability [7], which may affect the accuracy and consistency of follow-up examinations and impact the radiological workflow and further clinical decision-making.

Despite the differences observed in the absolute values based on different subgroups, by demonstrating strong correlation and agreement between the automated and manual measurements of the aortic diameter in a patient population with various aortic pathologies, this study indicates the high potential of the investigated algorithm to accelerate and standardize this complex and tedious assessment. With a median systematic error of −0.1 mm and a mean bias of −1.3 mm, the differences between the automated and manual measurements are comparable to those reported between different radiologists [13,14]. These results are not only consistent but even more promising regarding their errors when compared to previous studies that have evaluated algorithms for automated assessment of aortic diameters [9,14,15,16,17,18,19,20,21]. While most of these studies have analyzed algorithms that allow automated quantification of the aortic diameter at predefined locations, the present study evaluated an algorithm that automatically measures the largest diameter of the entire thoracic aorta rather than limiting its analysis to pre-defined segments. This could be particularly beneficial, for example, when the algorithm is used on scans performed for other indications, directly highlighting the maximum extent of aortic dilation as an incidental finding, thereby preventing missed findings.

Despite the overall promising results in this heterogeneous population, there are several aspects that may impact the algorithm’s accuracy. However, our subgroup analysis showed little, if any, influence on the performance based on anatomic location, BMI or history of prior aortic repair and/or dissection. When using non-enhanced scans, the automated measurements showed a constant tendency toward overestimation compared to the manual values in every subgroup. This is most likely due to the fact that in non-enhanced scans, the distinction between the vessel lumen and the surrounding and/or pathological structures is limited; thus, the algorithm mainly measures the external vessel diameter [16], without being influenced by other changes. Accordingly, the subgroup analysis revealed minor differences regarding the algorithm’s performance only when using contrast-enhanced scans. First, slightly lower errors and biases in the automated measurements of the ascending aorta compared to those of the descending aorta were most likely due to the relatively tubular and straight structure of the ascending aorta, which is easier to segment than the more curved descending aorta, possibly affecting the reproducibility of measurements perpendicular to the centerline. Furthermore, in this study population, aortic pathologies were more common in the descending aorta, possibly limiting the algorithm’s performance. Second, the algorithm’s accuracy was slightly higher in obese than in non-obese patients, which may be have been due to the better delineation of the surrounding structures and the overall larger aortic diameter in those patients. Third, prior aortic repair and/or the presence of aortic dissection resulted in slightly higher rates of errors and bias in the automated compared to the manual values, with a tendency for systematic underestimation of the aortic diameters in the affected patients. This is in line with the results of Monti et al., who showed that aortic pathologies limit the accuracy of automated aortic diameter measurements [16]. Nevertheless, in all these scenarios, independent of the anatomical aortic segment used, the patients’ weight or their history of aortic repair and/or presence of aortic dissection, the AI-based automated measurements showed strong correlation and excellent agreement with the corresponding manual values. Furthermore, this DNN-based algorithm additionally detects aortic dissection with high accuracy, comparable to the performance shown for other algorithms [22,23]. This prompt detection may reduce delayed or missed diagnosis of aortic dissection, thus possibly preventing adverse consequences for affected patients [24].

The present study is novel in several aspects, including the assessment of the thoracic aortic diameter at the most dilated location and the additional detection of aortic dissection in a large and diverse dataset of non- and contrast-enhanced CT images, which reflects a real-world clinical scenario. Furthermore, by performing a subgroup analysis, the algorithm’s performance was tested even in possibly more challenging scenarios. By overall demonstrating the algorithm’s ability to consistently estimate aortic diameters and to reliably detect aortic dissections even in a heterogeneous population, this study indicates its potential in supporting the radiology workflow, detecting aortic dilation and dissection in an early and advanced stage and enabling precise patient monitoring in follow-up examinations, even after aortic repair.

There are some limitations to this study that should be acknowledged. First, the data were analyzed retrospectively and obtained from a single institution, which may limit the generalizability of our results to other clinical scenarios or populations. Second, its impact on the time-effectiveness, clinical outcome and cost-effectiveness of aortic aneurysm/dissection management was not evaluated and should be part of further studies. Third, this study did not investigate aneurysm detection within a general patient population. Fourth, this study did not compare the algorithm’s performance to other state-of-the-art methods.

In conclusion, this DNN algorithm enables accurate automated aortic diameter quantification as well as reliable detection of aortic dissection in a diverse patient population with various aortic pathologies. Its effects on the clinical workflow in daily routine and its cost-effectiveness should be investigated in further studies. Nevertheless, this study already indicates the algorithm’s potential to be a valuable tool in aiming at more efficient and precise assessment of aortic diseases in clinical practice.

## Figures and Tables

**Table 1:** Patient characteristics.

Clinical Characteristics		Total (*n* = 100)
Age (years)		67.0 (55.3/73.0)
Female		40 (40.0)
Body height (cm)		173.0 ± 10.4
Body weight (kg)		84.7 ± 23.1
BMI (kg/m^2^)		27.9 ± 6.1
Aortic dilation	Predominantly ascending	44 (44.0)
	Predominantly descending	56 (56.0)
History of prior aortic repair	Overall	56 (56.0)
	Surgical	29 (29.0)
	Endovascular	17 (17.0)
	Both	10 (10.0)
Aortic dissection		42 (42.0)

Values are mean ± standard deviation, median (interquartile range) or n (%). BMI = body mass index.

**Table 2:** Comparison between manual and automated aortic diameter quantification overall as well as in the different subgroups.

	Manual d (mm)	Automated d (mm)	Wilcoxon #	r #	ICC #	Bias #	LoA #
Overall							
	40.9 (37.3–48.2)	42.9 (38.9–50.0) †	<0.05	0.86	0.93	1.5	−7.8/10.8
		40.3 (36.6–46.9) ‡	<0.05	0.89	0.94	−1.3	−9.8/7.2
Subgroup I							
Ascending aorta	39.5 (37.3–43.5)	39.8 (37.4–43.9) ‡	0.075	0.84	0.92	0.3	−5.3/5.9
Descending aorta	44.0 (36.9–52.5)	40.8 (34.1–50.0) ‡	<0.05	0.94	0.94	−3.0	−12.7/6.7
Subgroup II							
BMI < 30 kg/m^2^	40.6 (37.3–48.3)	39.8 (36.3–46.8) ‡	<0.05	0.87	0.93	−1.5	−10.6/7.6
BMI ≥ 30 kg/m^2^	42.1 (37.6–48.6)	40.4 (37.1–47.5) ‡	0.113	0.93	0.95	−1.2	−8.3/5.9
Subgroup III							
Without aortic repair	38.9 (33.9–44.8)	39.1 (34.9–44.6) ‡	0.933	0.95	0.96	−0.8	−7.7/6.0
With aortic repair	43.0 (39.0–50.5)	43.0 (36.8–48.2) ‡	<0.05	0.84	0.91	−1.7	−11.2/7.8
Subgroup IV							
Without dissection	40.9 (35.7–48.5)	41.1 (37.1–46.8) ‡	0.352	0.94	0.94	−1.1	−10.1/7.9
With dissection	41.4 (37.7–48.2)	39.0 (36.5–46.9) ‡	<0.05	0.82	0.93	−1.6	−9.4/6.2

# Comparison between manual and automated measurements. † Measured in the non-contrast scan. ‡ Measured in the contrast-enhanced scan. d: diameter; ICC: intraclass correlation coefficient; LoA: limit of agreement.

## Data Availability

The data presented in this study are available on reasonable request from the corresponding author.
